# Novel Mechanism of Action on Hedgehog Signaling by a Suppressor of Fused Carboxy Terminal Variant

**DOI:** 10.1371/journal.pone.0037761

**Published:** 2012-05-29

**Authors:** Ulrica Tostar, Csaba Finta, Mohammed Ferdous-Ur Rahman, Takashi Shimokawa, Peter G. Zaphiropoulos

**Affiliations:** Department of Biosciences and Nutrition, Karolinska Institutet, Huddinge, Sweden; Karolinska Institutet, Sweden

## Abstract

The Suppressor of Fused (SUFU) protein plays an essential role in the Hedgehog (HH) signaling pathway, by regulation of the GLI transcription factors. Two major isoforms of human SUFU are known, a full-length (SUFU-FL) and a carboxy-terminal truncated (SUFU- ΔC) variant. Even though SUFU- ΔC is expressed at an equivalent level as SUFU-FL in certain tissues, the function of SUFU-ΔC and its impact on HH signal transduction is still unclear. In two cell lines from rhabdomyosarcoma, a tumor type associated with deregulated HH signaling, SUFU-ΔC mRNA was expressed at comparable levels as SUFU-FL mRNA, but at the protein level only low amounts of SUFU-ΔC were detectable. Heterologous expression provided support to the notion that the SUFU-ΔC protein is less stable compared to SUFU-FL. Despite this, biochemical analysis revealed that SUFU-ΔC could repress GLI2 and GLI1ΔN, but not GLI1FL, transcriptional activity to the same extent as SUFU-FL. Moreover, under conditions of activated HH signaling SUFU-ΔC was more effective than SUFU-FL in inhibiting GLI1ΔN. Importantly, co-expression with GLI1FL indicated that SUFU-ΔC but not SUFU-FL reduced the protein levels of GLI1FL. Additionally, confocal microscopy revealed a co-localization of GLI1FL with SUFU-ΔC but not SUFU-FL in aggregate structures. Moreover, specific siRNA mediated knock-down of SUFU-ΔC resulted in up-regulation of the protein levels of GLI1FL and the HH signaling target genes *PTCH1* and *HHIP*. Our results are therefore suggesting the presence of novel regulatory controls in the HH signaling pathway, which are elicited by the distinct mechanism of action of the two alternative spliced SUFU proteins.

## Introduction

The Hedgehog (HH) signaling pathway is fundamental during embryonic development, and is also implicated in the growth of a variety of tumors [1]. The HH proteins are secreted ligands, which bind to the Patched 1 (PTCH1) receptor and initiate additional signaling events, ultimately resulting in the activation of the GLI transcription factors. The intracellular protein Suppressor of Fused (SUFU) acts as a negative regulator in the pathway, by binding to the GLI factors in the absence of signal activity and thereby repressing their transcriptional effects [2]. SUFU sequesters GLI1 in the cytoplasm but can also bind to GLI1 on DNA and actively export the nuclear GLI1 protein [3]. Additionally, SUFU binds to the other two GLI proteins, GLI2 and GLI3, and has recently been shown to be an important regulator of GLI2 and GLI3 processing, promoting the production of the truncated repressors [4], [5]. HH stimulation triggers the dissociation of SUFU-GLI complexes [4], [6]. The mechanism whereby the HH signal counteracts the repression of SUFU on GLI activity may also involve the ubiquitin-proteasome pathway-mediated degradation of SUFU [7].

Loss of function of mammalian SUFU leads to ligand-independent activation of the HH pathway [8]. Mutations in human *SUFU* have been found in medulloblastoma [9] and prostate cancer [10], and *SUFU* loss of heterozygosity was observed in rhabdomyosarcoma (RMS) [11], implicating that *SUFU* is a tumor suppressor gene.

**Figure 1:**
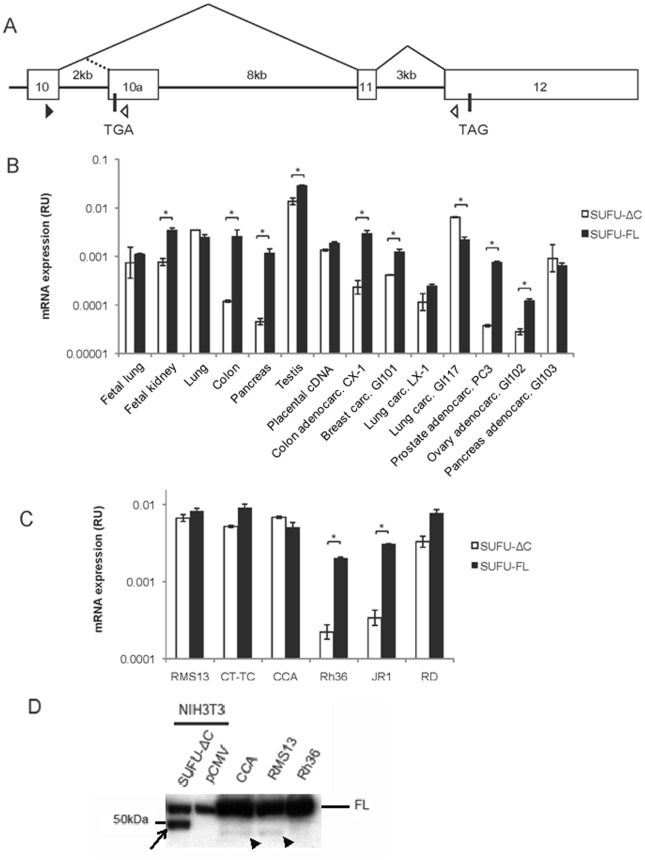
Expression of SUFU-FL and SUFU-ΔC in human tissues and cell lines. **A**, Schematic map of the SUFU exon 10 to exon 12 genomic region. Exons are shown by open boxes and splicing events by thin lines. The translation termination codons TGA and TAG are indicated, while the black and white triangles show the position of the PCR primers used. **B**, Real-time PCR analysis of SUFU-FL and SUFU-ΔC expression in a multiple tissue/tumor cDNA panel. **C**, Real-time RT-PCR analysis of SUFU-FL and SUFU-ΔC expression in the E-RMS cell lines JR-1, RD, Rh36, CCA, CT-TC and the A-RMS cell line RMS13. For both the B and C panels the data are presented as relative unit (RU) expression values, after normalization to the housekeeping gene large ribosomal protein (RPLPO), which is given the value of 1. Error bars indicate the standard deviation. *, Statistical significant difference, p<0,01, (Student’s *t* test). **D**, Western blot analysis of extracts from NIH3T3, transfected with an expression construct for SUFU-ΔC-FLAG, or pCMV (vector), CCA, RMS13 and Rh36 cells, and detected with a SUFU antibody. Note that CCA and RMS13 but not Rh36 cells express a protein band (arrowheads), which co-migrates with that of the transfected SUFU-ΔC FLAG-tagged construct (arrow), the small size difference apparently reflecting the presence of a FLAG tag. The endogenous SUFU-FL protein is indicated by FL.

Human *SUFU* is localized on chromosome 10q24–25, and contains 12 exons [12]. In addition to the full-length 484 amino acids (aa) protein, SUFU-FL, several alternatively spliced variants of SUFU have previously been identified. One of them codes for a carboxy-terminal deleted isoform of 433 aa, reducing therefore the calculated molecular mass from 54 to 48 kDa [13]. The truncated SUFU, SUFU-ΔC, uses a unique terminal exon harboring an early in-frame translation termination codon, exon 10a, which is derived from sequences within intron 10 of *SUFU* (Fig 1A).

Most studies have focused on SUFU-FL, while the function of SUFU-ΔC remains unclear. The aim of this work has been to analyze the biological properties of SUFU-ΔC and its implications on the transduction of the HH signal. These experimental approaches provide now support for a functional role of SUFU-ΔC and have resulted in the identification of novel mechanisms through which this SUFU variant can regulate the activity of the GLI transcription factors.

## Results

### Expression of SUFU Splice Variants

We first investigated the expression of SUFU in normal tissue and cancer cell lines by real-time RT-PCR (Fig 1B). SUFU-ΔC was generally expressed at lower levels than SUFU-FL, however in the lung comparable amounts of the variants were detected and in the lung cancer cell line GI117 higher levels of SUFU-ΔC relative to SUFU-FL could be observed. We also evaluated the expression pattern in RMS, a tumor associated with deregulated HH signaling [11], [14]. A variable expression was seen among the RMS cell lines, with most having a higher expression of SUFU-FL than SUFU-ΔC, however in CCA and RMS13 cells the levels were comparable (Fig 1C). To investigate whether the SUFU-ΔC protein can be detected, we first transfected NIH3T3 cells with an expression construct of SUFU-ΔC followed by whole cell protein extractions. On Western blot analysis a protein band of the expected 48 kDa size was observed with the SUFU-ΔC transfected construct, which was absent with the empty vector (Fig 1D). To examine whether it was possible to detect endogenous levels of SUFU-ΔC, the RMS13, CCA and Rh36 cell lines were also used. In contrast to the abundant protein expression of SUFU-FL in these cells, low levels of a protein band co-migrating with SUFU-ΔC at the 48 kDa region were detected in the RMS13 and CCA cell lines (Fig 1D). Interestingly, in the Rh36 cells, which have reduced SUFU-ΔC mRNA expression, no SUFU-ΔC protein was observed. Thus, even though the mRNA levels of the SUFU variants in either RMS13 or CCA cells are similar, the amounts of the corresponding proteins are dissimilar, with only low levels of SUFU-ΔC detected.

### Expression of SUFU-ΔC and SUFU-FL Constructs in Hek293 Cells

**Figure 2:**
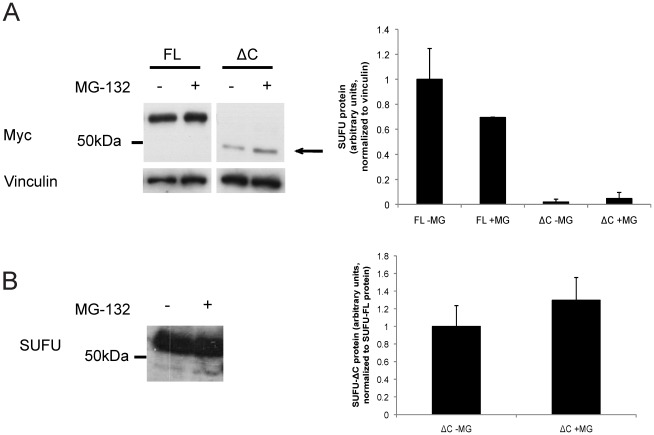
Impact of proteasome inhibition on exogenous and endogenous SUFU. A , Western blot analysis of soluble protein fractions from Hek293 cells, transfected with expression constructs for Myc-SUFU-FL (FL) or Myc-SUFU-ΔC (ΔC) and treated with or without 10 µM MG-132 for 6 hours. A Myc antibody was used for protein detection. Vinculin staining was used as a loading control. Note the higher levels of SUFU-FL protein relative to that of SUFU-ΔC, which is shown by an arrow. **B**, Western blot analysis of extracts from RMS13 cells with or without 10 µM MG-132 treatment for 6 hours. A SUFU antibody was used for protein detection. Note the increase of the lower protein band (SUFU-ΔC) relative to the upper protein band (SUFU-FL). For both the A and B panels duplicate experiments were performed and a quantification of the average protein levels is shown to the right.

To examine whether the differences in endogenous protein levels of the SUFU variants in the human RMS cell lines are also reflected on exogenously added proteins, Hek293 cells were transfected with SUFU-FL and SUFU-ΔC expression constructs. Indeed, the steady state levels of the exogenous SUFU variants were in line with what has been observed for the endogenous proteins, namely, reduced amounts of SUFU-ΔC relative to SUFU-FL. Additionally, treatment of the transfected cells with the proteasome inhibitor MG-132 conferred a detectable increase in the expression levels of the SUFU-ΔC (Fig 2A). Similarly, the endogenous SUFU-ΔC protein in RMS13 cells was also increased by MG-132 treatment (Fig 2B). Worth noting is that expression of the two isoforms in *E.coli* [15] revealed much higher levels of SUFU-FL than SUFU-ΔC in the soluble fraction (Figure S1). In conclusion, the heterologous expression analysis of the SUFU variants in the human Hek293 cell line is in agreement with the observations of low endogenous protein levels of SUFU-ΔC compared with SUFU-FL in the human RMS cells.

### GLI repression by SUFU-ΔC: Reduced Activity Against GLI1FL but not GLI1ΔN or GLI2

**Figure 3:**
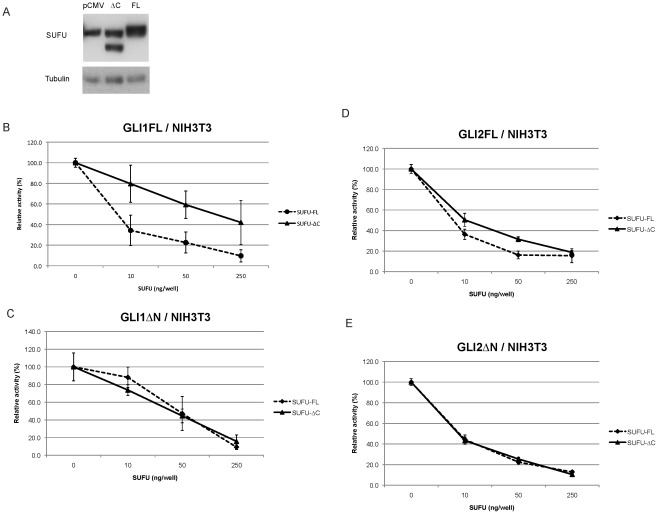
Dose-dependent SUFU repression of GLI activity in NIH3T3 cells. A . Western blot analysis of extracts from NIH3T3 cells, transfected with expression constructs for the SUFU variants, and detected with a SUFU antibody. Due to the partial co-migration with endogenous mouse Sufu, the SUFU-FL expression in the right lane was calculated by subtracting from the total signal the mouse Sufu signal. This was determined based on the Sufu expression in the left lane following normalization with the tubulin internal control. Note that the quantitation revealed that the level of SUFU-FL (right lane, upper protein band) is 188% the level of SUFU-ΔC (middle lane, lower protein band). **B–E**, Different amounts of Myc-tagged SUFU-FL or SUFU-ΔC expression constructs were co-transfected with 50 ng GLI expression constructs (Panel **B**, GLI1FL; panel **C**, GLI1ΔN; panel **D**, GLI2FL; panel **E**, GLI2ΔN), 12xGLIBS-luc and pRL-SV reporter plasmids, and the luciferase activity was measured. Error bars indicate the standard deviation.

Despite the lower protein expression of SUFU-ΔC relative to SUFU-FL, we set on to address the possible repressive function of SUFU-ΔC on GLI activity by reporter assays in NIH3T3 cells. In this mouse embryonic fibroblast cell line the protein expression of the SUFU-ΔC and SUFU-FL constructs is not as different as seen in Hek293 cells (Fig 3A). SUFU and GLI1 are known to bind to each other in the N-terminal and in the C-terminal regions of both proteins [16], [17]. As expected, SUFU-FL was capable of effectively repressing full length GLI1 (GLI1FL) transcriptional activity; on the other hand SUFU-ΔC could not act as an equally efficient repressor (Fig 3B). This suggests a role of the last 51 amino acids of SUFU in partially mediating the repression on GLI1FL. However, when a GLI1 variant lacking the N-terminal region, GLI1ΔN [18], was used, the repression elicited by either SUFU-FL or SUFU-ΔC was comparable (Fig 3C).

Additionally, for GLI2 and the N-terminal-truncated active form, GLI2ΔN, no major differences between the SUFU-FL and SUFU-ΔC repressive effects could be observed (Fig 3D and E).

### Effect of Introduction of SUFU-ΔC into *Sufu−/−* MEFs

**Figure 4 pone-0037761-g004:**
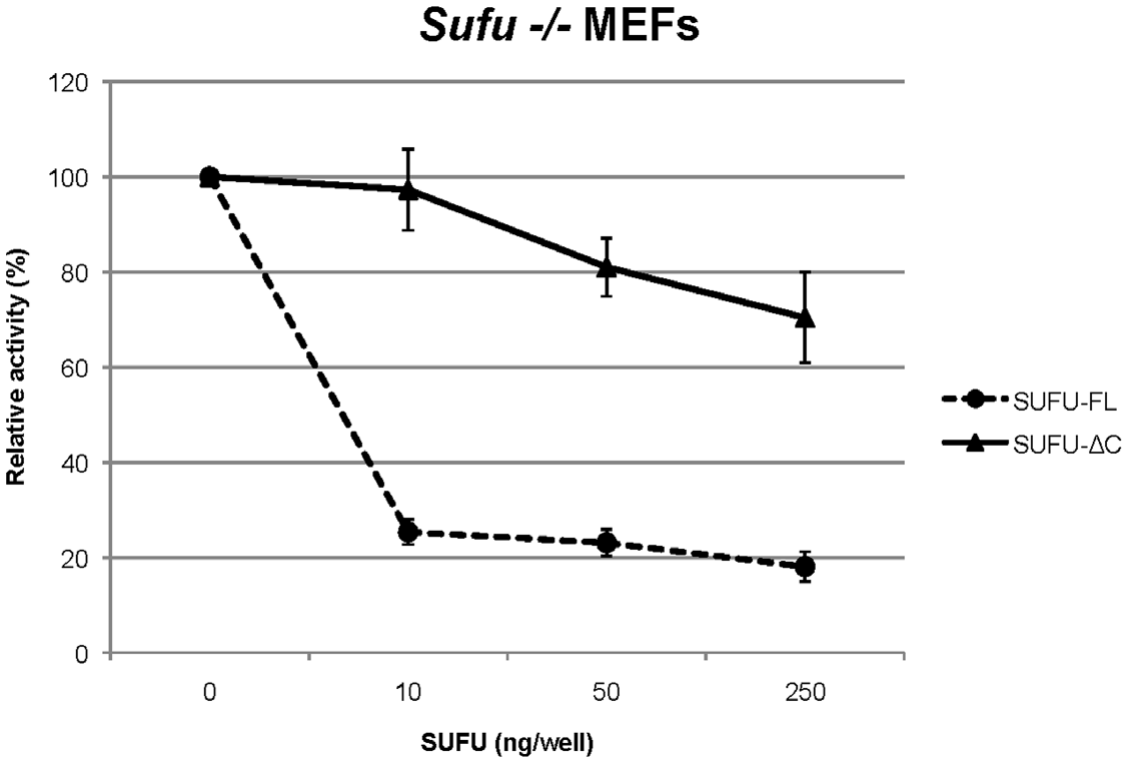
Dose-dependent SUFU repression of GLI activity in *Sufu−/−* MEFs. Different amounts of SUFU-FL or SUFU-ΔC FLAG-tagged expression constructs were co-transfected with 8xGLIBS-luc and pRL-SV reporter plasmids, and the luciferase activity was measured. Error bars indicate the standard deviation. NS, non-specific; co-transfection with 8xmutatedGLIBS-luc.

To further evaluate the functional capacity of SUFU-ΔC in relation to SUFU-FL we introduced the two expression constructs into mouse embryonic fibroblasts lacking Sufu (*Sufu−/−* MEFs), together with a GLI reporter construct. SUFU-FL was found to have a stronger effect than SUFU-ΔC in reducing GLI activity (Fig 4). Possibly, this may reflect the increased ratio of Gli1 to Gli2 in *Sufu−/−* relative to wild type MEFs [8], which would be in line with the over-expression experiments of Fig 3B and D.

### HH Signal Dependency of SUFU Inhibitory Activity

**Figure 5:**
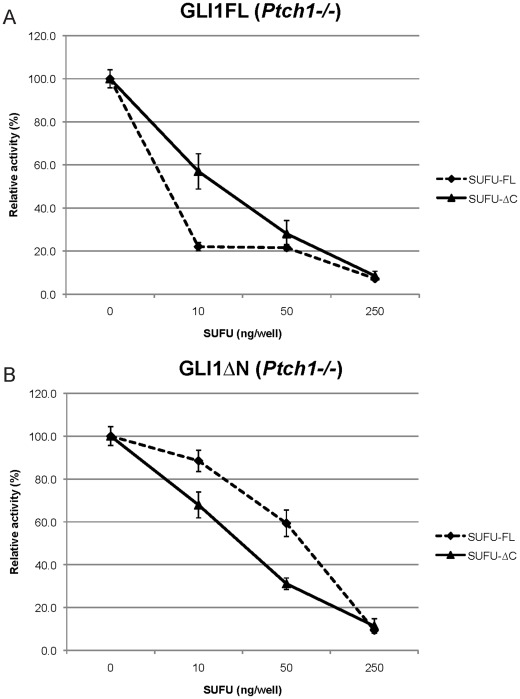
Dose-dependent SUFU repression of GLI1 activity in constitutively active *Ptch1−/−* MEFs. Different amounts of Myc-tagged SUFU-FL or SUFU-ΔC expression constructs were co-transfected with 50 ng GLI1 expression constructs (Panel **A**, GLI1FL; panel **B**, GLI1ΔN), 12xGLIBS-luc and pRL-SV reporter plasmids, and the luciferase activity was measured. Error bars indicate the standard deviation. Note the increased capacity of SUFU-ΔC relative to SUFU-FL in inhibiting GLI1ΔN transcriptional activity.

We also investigated whether HH signaling activation may modulate the SUFU repressive effects on GLI1. For this purpose mouse embryonic fibroblast cells lacking Ptch1 (*Ptch1−/−* MEFs), and therefore characterized by a constitutively active HH signaling pathway, were used. The results indicated that the maximal repression was observed with SUFU-FL acting on GLI1FL, as seen with NIH3T3 cells. SUFU-ΔC, on the other hand, was found to be more effective than SUFU-FL in repressing the GLI1ΔN variant (Fig 5A and B).

### Over-expression of SUFU-ΔC but not SUFU-FL Elicits a Reduction in GLI1FL

**Figure 6:**
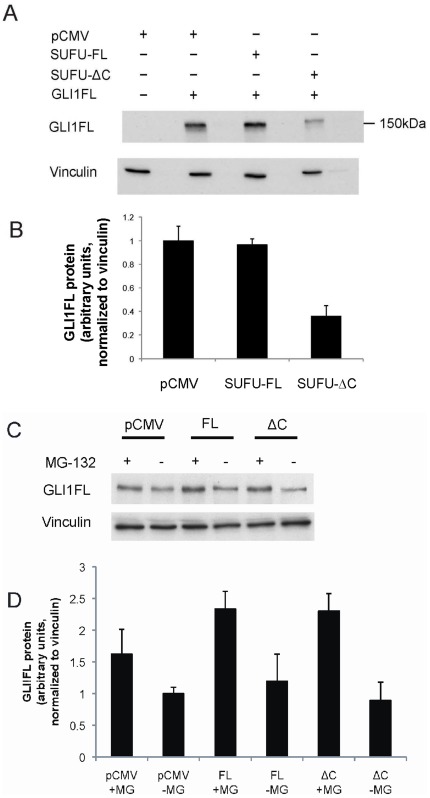
SUFU-ΔC but not SUFU-FL reduces GLI1FL protein levels. A , GLI1FL was introduced into Hek293 cells together with either SUFU-FL or SUFU-ΔC FLAG-tagged expression constructs, as indicated. Soluble protein fractions were run on a SDS-PAGE gel, followed by Western blotting, and detected by a GLI1 antibody. Vinculin staining was used as a loading control. **B**, Quantification of average GLI1FL protein levels in this and in an additional repeat experiment. Note that SUFU-ΔC but not SUFU-FL elicits a reduction of GLI1FL protein. **C**, SUFU-FL (FL) or SUFU-ΔC (ΔC) was introduced into RMS13 cells, with or without 10 µM MG-132 treatment for 6 hours. Soluble protein fractions were run on a SDS-PAGE gel, followed by Western blotting, and detected by a GLI1 antibody. Vinculin staining was used as a loading control. **D**, Quantification of average GLI1FL protein levels in this and in an additional repeat experiment. Note that SUFU-ΔC but not SUFU-FL elicits a detectable reduction of GLI1FL protein and that MG-132 treatment confers an increase in GLI1FL protein.

Since the SUFU variants had differential effects on the GLI1FL transcriptional activity we examined their impact on the GLI1FL protein levels. Surprisingly, co-expression of GLI1FL with SUFU-ΔC in Hek293 cells resulted in lower amounts of GLI1FL relative to co-expression with either SUFU-FL or the empty vector (Fig 6A and B). These findings suggest that the mechanism of inhibition of GLI1FL activity by SUFU-ΔC may be fundamentally different to the one elicited by SUFU-FL, and could involve an increased degradation of the GLI1FL protein. Interestingly, the levels of GLI2 remained unchanged in the same transfection setting (Figure S2), highlighting the specificity of the GLI1FL and SUFU-ΔC interaction. Additionally, the levels of endogenous GLI1FL in RMS13 cells were investigated following transfection of the SUFU variants. SUFU-ΔC, in contrast to SUFU-FL, conferred a detectable reduction of the GLI1FL levels. This reduction is not as high as seen with exogenous GLI1FL in Hek293, apparently the result of the relatively low RMS13 transfection efficiency (usually less than 50%), which allows the presence of a large number of untransfected cells that express high levels of GLI1FL. Moreover, the proteasome inhibitor MG-132 increased the GLI1FL protein in both SUFU-ΔC and SUFU-FL transfected cells. (Fig 6C and D).

### Subcellular Localization of SUFU-ΔC

**Figure 7:**
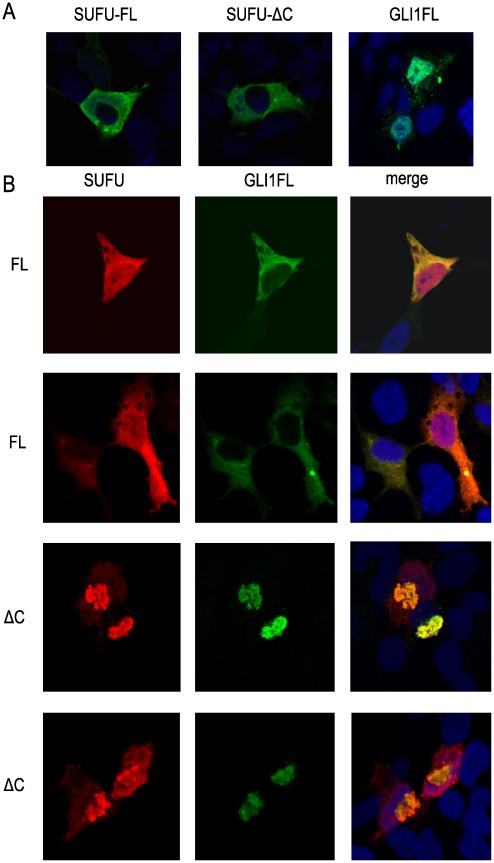
Subcellular localization of SUFU-FL and SUFU-ΔC in Hek293 cells. The transfected SUFU and/or GLI1FL constructs were detected using Myc and FLAG antibodies by confocal microscopy. **A**, Individual transfections of Myc-tagged SUFU-FL or SUFU-ΔC and FLAG-tagged GLI1FL constructs. **B**, Co-transfections of GLI1FL with the SUFU-FL (FL) or the SUFU-ΔC (ΔC) construct. Two representative images from each co-transfection are shown. The nuclei are stained with the marker DRAQ5 (blue signal). Note that GLI1FL co-localizes with SUFU-ΔC in aggregate/clump structures in the cytoplasm.

Confocal microscopy with Hek293 cells was utilized to examine the subcellular localization of transfected SUFU-FL and SUFU-ΔC. In line with previous reports [19], [20] SUFU-FL had a predominant cytoplasmic localization, and we also found SUFU-ΔC to be mainly detected in the cytoplasm (Fig 7A, left and middle panel). GLI1FL was detected in the nucleus (Fig 7A, right panel), as shown previously [17], but there were several cases where a cytoplasmic localization was also observed. Co-expression of GLI1FL and SUFU-FL resulted in co-localization in the cytoplasm (Fig 7B, upper panels). SUFU-ΔC also co-localized with GLI1FL, however, predominantly in aggregate/clump structures in close proximity to the nucleus (Fig 7B, lower panels).

### Knock-down of Endogenous SUFU-ΔC Increases GLI1FL Protein Levels and Up-regulates HH Signaling Targets

**Figure 8:**
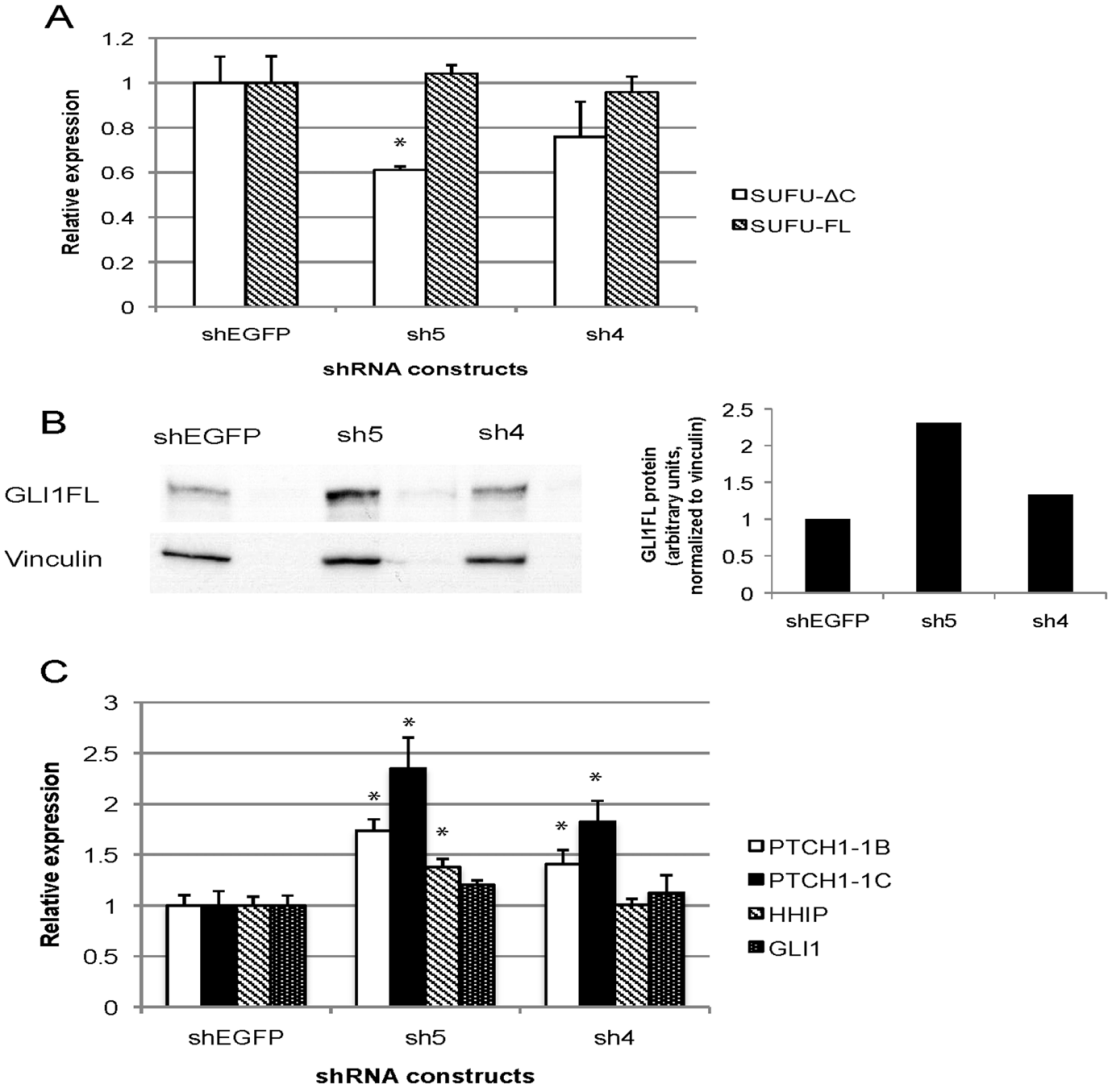
Knock-down of SUFU-ΔC expression in RMS13 cells results in increased GLI1FL protein and up-regulation of HH targets. **A**, Real-time RT-PCR analysis of the relative expression of SUFU-ΔC and SUFU-FL following transfection of the sh4, sh5 or shEGFP construct. **B**, Western blot analysis of soluble protein fractions with a GLI1 antibody following transfection of the sh4, sh5 or shEGFP construct. A quantification of the protein levels is shown to the right. **C**, Real-time RT-PCR analysis of the relative expression of HHIP, GLI1, PTCH1-1B and PTCH1-1C following transfection of the sh4, sh5 or shEGFP construct. In both the A and C panels the expression levels relative to the EGFP control, normalized to the mean expression of the housekeeping genes RPLPO and TBP, are shown. Error bars indicate the standard deviation. *, Statistical significant, p<0,05, compared with control (Student’s *t* test). Note that the more effective the knock-down of SUFU-ΔC, the higher the increase of GLI1FL protein and the expression of HH signaling target genes.

To investigate the role of endogenous SUFU-ΔC, two shRNA constructs targeting SUFU-ΔC (sh4 and sh5) were individually introduced into the RMS13 cell line. Transfection of the shRNAs did not alter the SUFU-FL expression levels, as determined by real-time RT-PCR analysis, highlighting their specificity for SUFU-ΔC (Fig 8A). Additionally, a detectable reduction of SUFU-ΔC protein was observed by sh5 (Figure S3), with the effect seen being less than that of the SUFU-ΔC mRNA. Subsequently, the GLI1 protein levels were analyzed by Western blotting. In line with the negative impact of SUFU-ΔC on the GLI1FL protein levels, observed in the analysis of Fig 6, the most effective siRNA used (sh5) resulted in increased amounts of GLI1FL protein (Fig 8B). Moreover, the HH signaling target gene *HHIP* and the HH signaling regulated transcript variants of the *PTCH1* gene, PTCH1-1B and PTCH1-1C [21], [22], were up-regulated by the shRNA treatments. Interestingly, the extent of up-regulation appeared to parallel the effectiveness of the two shRNAs in down-regulating SUFU-ΔC. Additionally, the GLI1 mRNA was not significantly changed by the introduction of the shRNAs (Fig 8C). Thus, our data provide evidence that endogenous SUFU-ΔC can modulate HH signaling activity through a mechanism that is distinct from that of SUFU-FL, namely by reducing GLI1FL protein levels.

## Discussion

Human SUFU plays an essential role during both normal development and cancerous transformation. SUFU is known to functionally regulate the GLI proteins, the ultimate effectors of the HH pathway [3]. However, the exact role of human SUFU-FL is still not fully deciphered, and even more so the function of the alternatively spliced variant SUFU-ΔC.

Although both SUFU isoforms could be detected at the mRNA level, only low levels of the endogenous SUFU-ΔC protein were observed in the Western blot analysis of the RMS cell lines. This indicates that the SUFU-ΔC is less stable than SUFU-FL. The analysis of the heterologous expression of the SUFU variants in the Hek293 cell line is in agreement with a reduced stability of SUFU-ΔC relative to SUFU-FL in human RMS cells. Moreover, treatment with the proteasome inhibitor MG-132 confers a selective increase of both the endogenous and exogenous SUFU-ΔC protein, however, the levels of SUFU-FL are not reached. Consequently, one has to hypothesize that other mechanisms may also have a role in the reduced steady state levels of SUFU-ΔC, possibly lysosomal autophagy as recently implicated in Wnt signaling components [23]. Interestingly, in the mouse embryonic fibroblast cell line NIH3T3, the protein levels of transfected SUFU-FL and SUFU-ΔC are not as pronouncedly different.

GLI2 is the primary activator of HH-dependent transcription. Our biochemical analysis in NIH3T3 cells showed that SUFU-ΔC could repress GLI2 transcriptional activity to the same extent as SUFU-FL. These observations are in line with an earlier study, which showed that a SUFU deletion construct lacking the amino acids 389–485 was fully capable to repress GLI2, but to a lesser extent GLI1 [20].In a previous study we have shown that GLI1 is the most important GLI factor in E-RMS [24]. Now we provide evidence that SUFU-FL has generally a higher capacity than SUFU-ΔC in inhibiting GLI1 activity. However, in certain cellular contexts SUFU-ΔC may have a major impact on the transcriptional activation elicited by GLI1. For example, our data (Fig 5) indicate that SUFU-ΔC is more effective than SUFU-FL in inhibiting the transcriptional activity of an amino terminal variant of GLI1, GLI1ΔN, during conditions of activated HH signaling. On the other hand in *Sufu−/−* MEFs, SUFU-FL elicited a stronger inhibition than SUFU-ΔC on GLI activity (Fig 4), possibly reflecting the increased amounts of Gli1 in these cells, which would be consistent with the data of Fig 3B.

Importantly, the co-expression experiments of GLI1FL with the SUFU variants suggest that distinct regulatory mechanisms act on GLI1FL. SUFU-FL does not alter the protein levels of GLI1FL but is quite effective in repressing its transcriptional activity. On the other hand SUFU-ΔC is a less effective transcriptional inhibitor, but elicits a reduction of the GLI1FL protein levels (Figs 3B and 6). Thus, SUFU-ΔC, in line with its intrinsically lower stability in human cells, may act as a scavenger by promoting GLI1FL degradation instead of only shuttling GLI1FL to the cytoplasm, as does SUFU-FL [3]. This scenario is supported by the immunofluorescence data in which GLI1FL tends to localize to punctate cytoplasmic densities when co-expressed with SUFU-ΔC (Fig 7).

Moreover, specific knock-down of the SUFU-ΔC resulted in increased protein levels of GLI1FL and activation of HH signaling target genes (Fig 8), providing support for an *in vivo* function of this variant as a regulator of the GLI1FL protein and consequently HH signal transduction. This finding indicates that the relatively weaker repression of GLI1FL activity by SUFU-ΔC, determined by the over-expression analyses of Figs. 3–5, may be augmented by the destabilization of the GLI1FL protein. Taken together, our experimental data suggest that the mechanism of action of SUFU-ΔC is fundamentally different from that of SUFU-FL, as highlighted by the distinct effects on GLI1FL protein/activity.

In conclusion, our results provide support for the presence of novel regulatory mechanisms within the HH signaling pathway, which are linked to the alternative splicing of SUFU. Further evaluation of the SUFU isoforms will be needed in order to unfold additional complexities of their differential function and regulation.

## Materials and Methods

### Cell Lines and Reagents

The NIH3T3 cell line, the Hek293 cell line, the alveolar rhabdomyosarcoma (A-RMS) cell line RMS13 and the embryonal rhabdomyosarcoma (E-RMS) cell line RD were purchased from ATCC (Manassas, VA, USA), the E-RMS cell lines JR-1 and Rh36 were kind gifts from P. Houghton (St. Jude Childrens Research Hospital, USA), CCA was a kind gift from P-L Lollini (University of Bologna, Italy), and CT-TC a kind gift from H. Hosoi (Kyoto Prefectural University of Medicine, Japan. *Ptch1−/−* MEFs was a kind gift from J. Taipale (University of Helsinki, Finland), and *Sufu−/−* mouse embryonic fibroblasts (MEFs) were established by S. Teglund in our laboratory [8]. cDNA panels (Human tissues and human tumor cell lines) were purchased from BD Biosciences (San Jose, CA, USA). MG-132 was purchased from Calbiochem (San Diego, CA, USA).

### Real-time RT-PCR

Total RNA from cells was prepared with the RNeasy kit (Qiagen, Hamburg, Germany) followed by cDNA synthesis with random (N6) primers (New England Biolabs, Ipswich, MA, USA) and Superscript II (Invitrogen, Carlsbad, CA, USA). Real-time RT-PCR was performed with Power SYBR Green (Applied Biosystems, Foster City, CA, USA) on a 7500 Fast real-time PCR system (Applied Biosystems) with the following primers used; SUFU–Forward: 5′-TGCCACTGAGGAGCATCCTTACGC; SUFU-FL–Reverse: 5′-TTCAGGCCAGCTGTACTCTTTGGGAAG; SUFU-ΔC–Reverse: 5′-TCAGGGCAGAAAGACGTTCCAGGAC. Additional primer sets are found in the Table S1. mRNA expression levels were normalized against the housekeeping genes *RPLPO* and *TBP*, and the relative units after assigning *RPLPO* or the average of *RPLPO* and *TBP* expression to 1 are shown. Individual experiments were performed at least in triplicate and the data from a representative experiment are shown. Statistical significance was evaluated using a two-tailed, unpaired Student’s *t* test and p<0,05 was considered statistically significant.

### Western Blot

Proteins were extracted and separated on 15% or 7% SDS-PAGE essentially as described [21]. SUFU was detected with a rabbit polyclonal antibody (C81H7), GLI1 with a rabbit polyclonal antibody, both from Cell Signaling (Danvers, MA, USA), FLAG with a mouse monoclonal antibody (M2) from Sigma-Aldrich (St. Louis, MO, USA), GLI2 with a rabbit polyclonal antibody from Aviva Systems Biology (San Diego, CA, USA) and vinculin with a mouse monoclonal antibody (V9131) from Sigma-Aldrich. Horseradish peroxidase-conjugated anti-rabbit IgG or anti-mouse IgG (GE Healthcare, Little Chalfont, Buckinghamshire, UK) was used as the secondary antibody, followed by detection of the proteins with the Western Lightning Western blot Chemiluminescence Reagent Plus (PerkinElmer Life Sciences, Waltham, MA, USA). Individual experiments were performed at least in duplicate and the data from a representative experiment are shown. Protein levels were quantified using the Fiji software (ImageJ/NIH).

### Expression Constructs and Transfection

Expression and reporter constructs have been described elsewhere [3], [18]. The SUFU-FL-EYFP was a kind gift from M. Lauth, the GLI2FL was a kind gift from E. Roessler and the GLI2ΔN was a kind gift from F. Aberger. The Myc-SUFU-ΔC expression construct was generated by replacement of the Xba1 and NspE1 digested Myc-SUFU-FL expression construct with a RT/PCR product amplified by the primer set 5′-GCTTCTAGAATAGGTTCAGAGTTGT and 5′-ACCAATCAACCCTCAGCGGCAGAAT. The SUFU-FL-FLAG and SUFU-ΔC-FLAG constructs were generated from the SUFU-FL-EYFP plasmid. Following BspE1/Xba1 double digestion, ligation of PCR products generated by the primer sets SUFU-Forward 5′-ACCAATCAACCCTCAGCGGCAGAAT and either FLAG-SUFU-FL-Reverse 5′-GCTTCTAGACTACTTGTCATCGTCGTCCTTGTAATCGTGTAGCGGACTGTCG or FLAG-SUFU-ΔC-Reverse 5′- GCTTCTAGACTACTTGTCATCGTCGTCCTTGTAATCGAGTTGTAACCAGGGT were performed. For all expression constructs the pCMV vector was used. The expression constructs and the appropriate reporter construct were transfected into cultured cells using FuGENE6 (Roche Diagnostics, Mannheim, Germany) or Lipofectamine 2000 (Invitrogen). Expression of the SUFU variants in *E.coli* is described in Methods S1.

### Reporter Assays

Luciferase reporter assays were performed essentially as described previously [18], [21]. All experiments were analyzed independently three times, and a representative experiment is shown.

### Immunofluorescence

Hek293 cells were plated in 4-well poly-d-lysine-coated dishes, and transfected 24 hours later with Myc-tagged SUFU constructs and GLI1FL-FLAG. 48 h after transfection, cells were fixed, permeabilized, incubated with anti-FLAG (Sigma-Aldrich) and/or anti-Myc (Santa Cruz; sc-789) overnight and fluorescently labeled with Alexa Fluor dye-conjugated secondary antibodies (Molecular Probes, Eugene, OR, USA). Imaging of the immunostained cells was performed using a Zeiss LSM510 confocal microscope. Individual experiments were performed at least in triplicate and the data from a representative experiment are shown.

### RNA Interference

Two shRNA constructs (sh4 and sh5) targeting SUFU-ΔC were designed using sequences from SUFU exon 10a and the Insert Design Tool for the pSilencer Vectors (Ambion, Austin, TX, USA). As a negative control, a shRNA targeting the Enhanced Green Fluorescent Protein (EGFP), shEGFP, was also designed. Complementary oligonucleotides with BamHI and HindIII sites at the ends were ordered from Sigma-Aldrich with the following sequences; sh4: 5′-CCTATCCTCGGAGCTCTGCTTCAAGAGAGCAGAGCTCCGAGGATAGGTT, sh5: 5′-TTTTCAGCAGCTCAAGAACTTCAAGAGAGTTCTTGAGCTGCTGAAAATT, shEGFP: 5′-GAAGCAGCACGACTTCTTCTTCAAGAGAGAAGAAGTCGTGCTGCTTCAT and cloned into the pSilencer 4.1-CMVpuro vector. All constructs were verified by sequencing. RMS13 cells were transfected with the shRNA constructs and subjected to puromycin (1 µg/mL) selection for 4 days, followed by RNA isolation and real-time RT-PCR.

## Supporting Information

Figure S1 **Heterologous expression of SUFU variants in** ***E. coli*** **.** SUFU-FL (upper panel) and SUFU-ΔC (lower panel) constructs were purified from E. coli as described above. Chromatograms of the last purification step, size-exclusion chromatography, are shown. Open arrows indicate peaks corresponding to protein eluted with the void volume (aggregated form), while filled arrows indicate peaks corresponding to monomeric (soluble form) protein fractions. Note the decreased amount of SUFU-ΔC relative to SUFU-FL in the soluble fraction.
(PDF)

Figure S2 **The Western blot of** **Fig 6A** **was stripped and incubated with a GLI2 antibody.** Note that the levels of endogenous GLI2 remain unchanged irrespective of the introduction of SUFU-ΔC or SUFU-FL.
(PDF)

Figure S3 **Western blot analysis of SUFU protein levels after shRNA down-regulation.** shEGFP, sh5 or sh4 constructs were transfected into RMS13 cells, followed by SDS-PAGE gel electrophoresis and Western blot analysis by a SUFU antibody. Both a short and a long exposure are shown. SUFU-ΔC is indicated by an arrow. Vinculin was used as a loading control. A quantification of the SUFU-ΔC protein levels relative to SUFU-FL is shown below the blot, revealing a 33% reduction of SUFU-ΔC by sh5 RNA treatment.
(PDF)

Methods S1 **Heterologous protein expression in** ***E.coli*** **and purification.**
(PDF)

Table S1 **Sequence of PCR primers.**
(PDF)

## References

[1] Teglund S, Toftgård R (2010). Hedgehog beyond medulloblastoma and basal cell carcinoma..

[2] Cheng SY, Yue S (2008). Role and regulation of human tumor suppressor SUFU in Hedgehog signaling.. Adv Cancer Res.

[3] Kogerman P, Grimm T, Kogerman L, Krause D, Unden AB (1999). Mammalian suppressor-of-fused modulates nuclear-cytoplasmic shuttling of Gli-1.. Nat Cell Biol.

[4] Humke EW, Dorn KV, Milenkovic L, Scott MP, Rohatgi R (2010). The output of Hedgehog signaling is controlled by the dynamic association between Suppressor of Fused and the Gli proteins.. Genes Dev.

[5] Wang C, Pan Y, Wang B (2010). Suppressor of fused and Spop regulate the stability, processing and function of Gli2 and Gli3 full-length activators but not their repressors.. Development.

[6] Tukachinsky H, Lopez LV, Salic A (2010). A mechanism for vertebrate Hedgehog signaling: recruitment to cilia and dissociation of SuFu-Gli protein complexes.. J Cell Biol.

[7] Yue S, Chen Y, Cheng SY (2009). Hedgehog signaling promotes the degradation of tumor suppressor Sufu through the ubiquitin-proteasome pathway.. Oncogene.

[8] Svärd J, Heby-Henricson K, Persson-Lek M, Rozell B, Lauth M (2006). Genetic elimination of Suppressor of fused reveals an essential repressor function in the mammalian Hedgehog signaling pathway.. Dev Cell.

[9] Taylor MD, Liu L, Raffel C, Hui CC, Mainprize TG (2002). Mutations in SUFU predispose to medulloblastoma.. Nat Genet.

[10] Sheng T, Li C, Zhang X, Chi S, He N (2004). Activation of the hedgehog pathway in advanced prostate cancer.. Mol Cancer.

[11] Tostar U, Malm CJ, Meis-Kindblom JM, Kindblom LG, Toftgard R (2006). Deregulation of the hedgehog signalling pathway: a possible role for the PTCH and SUFU genes in human rhabdomyoma and rhabdomyosarcoma development.. J Pathol.

[12] Grimm T, Teglund S, Tackels D, Sangiorgi E, Gurrieri F (2001). Genomic organization and embryonic expression of Suppressor of Fused, a candidate gene for the split-hand/split-foot malformation type 3.. FEBS Lett.

[13] Stone DM, Murone M, Luoh S, Ye W, Armanini MP (1999). Characterization of the human suppressor of fused, a negative regulator of the zinc-finger transcription factor Gli.. J Cell Sci 112 (Pt.

[14] Gorlin RJ (2004). Nevoid basal cell carcinoma (Gorlin) syndrome.. Genet Med.

[15] Ekblad CM, Wilkinson HR, Schymkowitz JW, Rousseau F, Freund SM (2002). Characterisation of the BRCT domains of the breast cancer susceptibility gene product BRCA1.. J Mol Biol.

[16] Merchant M, Vajdos FF, Ultsch M, Maun HR, Wendt U (2004). Suppressor of fused regulates Gli activity through a dual binding mechanism.. Mol Cell Biol.

[17] Dunaeva M, Michelson P, Kogerman P, Toftgard R (2003). Characterization of the physical interaction of Gli proteins with SUFU proteins.. J Biol Chem.

[18] Shimokawa T, Tostar U, Lauth M, Palaniswamy R, Kasper M (2008). Novel human glioma-associated oncogene 1 (GLI1) splice variants reveal distinct mechanisms in the terminal transduction of the hedgehog signal.. J Biol Chem.

[19] Ding Q, Fukami S, Meng X, Nishizaki Y, Zhang X (1999). Mouse suppressor of fused is a negative regulator of sonic hedgehog signaling and alters the subcellular distribution of Gli1.. Curr Biol.

[20] Barnfield PC, Zhang X, Thanabalasingham V, Yoshida M, Hui CC (2005). Negative regulation of Gli1 and Gli2 activator function by Suppressor of fused through multiple mechanisms.. Differentiation.

[21] Shimokawa T, Rahnama F, Zaphiropoulos PG (2004). A novel first exon of the Patched1 gene is upregulated by Hedgehog signaling resulting in a protein with pathway inhibitory functions.. FEBS Lett.

[22] Shimokawa T, Svärd J, Heby-Henricson K, Teglund S, Toftgård R (2007). Distinct roles of first exon variants of the tumor-suppressor Patched1 in Hedgehog signaling.. Oncogene.

[23] Gao C, Cao W, Bao L, Zuo W, Xie G (2010). Autophagy negatively regulates Wnt signalling by promoting Dishevelled degradation.. Nat Cell Biol.

[24] Tostar U, Toftgård R, Zaphiropoulos PG, Shimokawa T (2010). Reduction of human embryonal rhabdomyosarcoma tumor growth by inhibition of the hedgehog signaling pathway.. Genes & Cancer.

