# Supplementary figures and images for: Novel Mechanism of Action on Hedgehog Signaling by a Suppressor of Fused Carboxy Terminal Variant
Source: PLoS One. 2012 May 29;7(5):e37761. doi: 10.1371/journal.pone.0037761 (PMC3362617; doi:10.1371/journal.pone.0037761)

## Supplemental fig 1

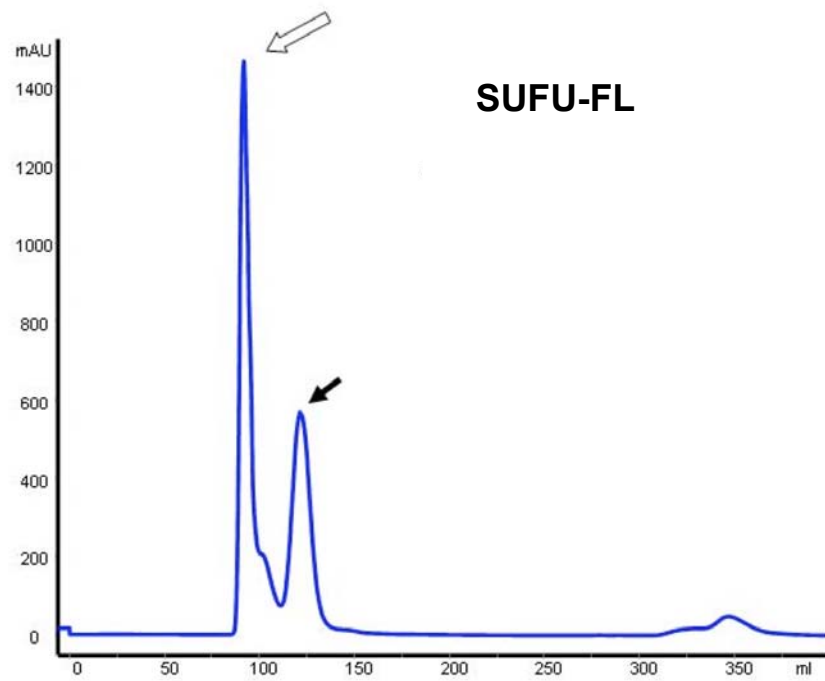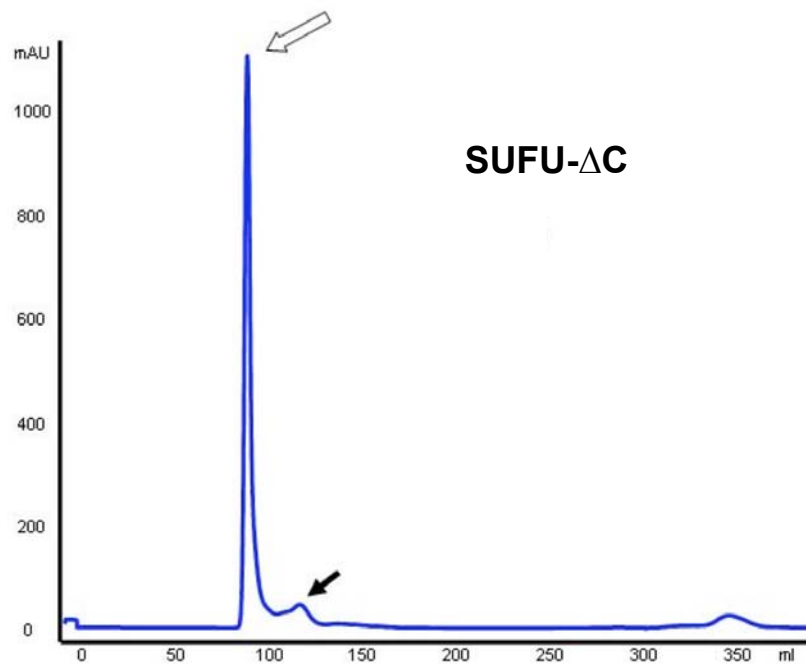

Supplement: Figure S1 — Heterologous expression of SUFU variants in E. coli . SUFU-FL (upper panel) and SUFU-ΔC (lower panel) constructs were purified from E. coli as described above. Chromatograms of the last purification step, size-exclusion chromatography, are shown. Open arrows indicate peaks corresponding to protein eluted with the void volume (aggregated form), while filled arrows indicate peaks corresponding to monomeric (soluble form) protein fractions. Note the decreased amount of SUFU-ΔC relative to SUFU-FL in the soluble fraction. (PDF) [file pone.0037761.s001.pdf]

## Supplemental fig 2

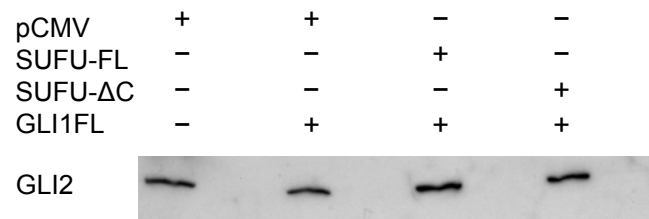

Supplement: Figure S2 — The Western blot of Fig 6A was stripped and incubated with a GLI2 antibody. Note that the levels of endogenous GLI2 remain unchanged irrespective of the introduction of SUFU-ΔC or SUFU-FL. (PDF) [file pone.0037761.s002.pdf]

### Supplemental fig 3

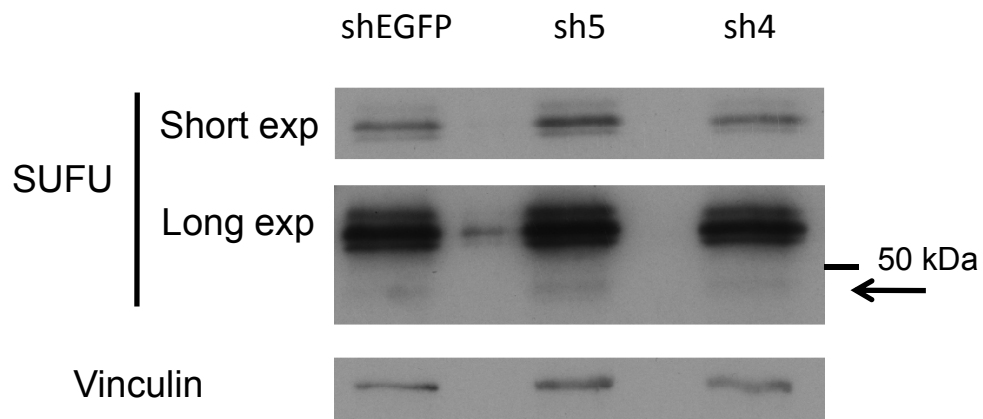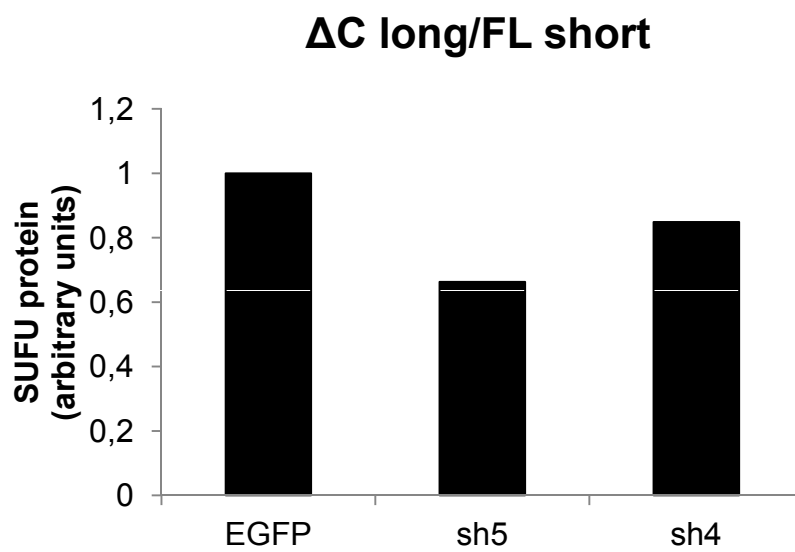

Supplement: Figure S3 — Western blot analysis of SUFU protein levels after shRNA down-regulation. shEGFP, sh5 or sh4 constructs were transfected into RMS13 cells, followed by SDS-PAGE gel electrophoresis and Western blot analysis by a SUFU antibody. Both a short and a long exposure are shown. SUFU-ΔC is indicated by an arrow. Vinculin was used as a loading control. A quantification of the SUFU-ΔC protein levels relative to SUFU-FL is shown below the blot, revealing a 33% reduction of SUFU-ΔC by sh5 RNA treatment. (PDF) [file pone.0037761.s003.pdf]
