# Supplementary material for: Novel Mechanism of Action on Hedgehog Signaling by a Suppressor of Fused Carboxy Terminal Variant
Source: PLoS One. 2012 May 29;7(5):e37761. doi: 10.1371/journal.pone.0037761 (PMC3362617; doi:10.1371/journal.pone.0037761)
Supplement: Methods S1 — Heterologous protein expression in E.coli and purification. (PDF) [file pone.0037761.s004.pdf]

Supplemental Materials and Methods

Heterologous protein expression in E.coli and purification

Detailed description of heterologous expression in E. coli and purification of SUFU proteins is to be described elsewhere (Finta et al, manuscript in preparation). Briefly, 6XHis tagged recombinant SUFU constructs were expressed in E. coli JM109 (DE3). Protein expression was induced using 0.1mM IPTG. Pelleted cells were lysed in 50mM Tris.Cl pH7.5, 50mM NaCl, 1mM MgCl2, 1mM DTT, 0.2mg/ml lysozyme, 25u/ml benzonase (Sigma-Aldrich) and Complete, Mini, EDTA-free protease inhibitor cocktail (Roche) using 3X freeze-thaw cycles. Lysate was cleared using centrifugation (18,000g for 30min), and the cleared lysate was loaded onto HisTrap HP column (GE Healthcare). Following extensive wash with 50mM Tris.Cl pH7.5, 1M NaCl, 20mM imidazole, 1mM DTT, bound proteins were eluted with 50mM Tris.Cl pH7.5, 50mM NaCl, 500mM imidazole, 1mM DTT. Concentrated eluate was loaded onto a HiPrep 26/60 Sephacryl S-200 HR gel-filtration column (GE Healthcare) and eluted in 10mM Tris.Cl pH7.4, 50mM NaCl and 1mM DTT.
