# Supplementary material for: Novel Mechanism of Action on Hedgehog Signaling by a Suppressor of Fused Carboxy Terminal Variant
Source: PLoS One. 2012 May 29;7(5):e37761. doi: 10.1371/journal.pone.0037761 (PMC3362617; doi:10.1371/journal.pone.0037761)
Supplement: Table S1 — Sequence of PCR primers. (PDF) [file pone.0037761.s005.pdf]

Supplemental Table

**Primer**	**5’ – 3’**
RPLPO-F	CCTTCTCCTTTGGGCTGGTCATCCA
RPLPO-R	CAGACACTGGCAACATTGCGGACAC
TBP-F	GCCAGCTTCGGAGAGTTCTGGGATT
TBP-R	CGGGCACGAAGTGCAATGGTCTTTA
PTCH1-1B-F	CCGCCTTCGCTCTGGAGCAGATT
PTCH1-1C-F	GTTGACGGCCGGCTATGGGGAAG
PTCH1-1B/1C-R	TCTGAAACTTCGCTCTCAGCCACAGC
HHIP-F	TTTTACACTTGCCGAGGCCATATTCCA
HHIP-R	AGCACAACCCACCATCTTTTCTTGCAT
GLI1-F	CAGCTACATCAACTCCGGCCAATAGGG
GLI1-R	TGCTGCGGCGTTCAAGAGAGACTG
